# A Nearly Missed Case of Severe Necrotizing Soft Tissue Infection

**DOI:** 10.7759/cureus.6856

**Published:** 2020-02-03

**Authors:** Andrea Sarchi, Brit Long

**Affiliations:** 1 Emergency Medicine, Brooke Army Medical Center, Fort Sam Houston, USA

**Keywords:** necrotizing soft tissue infection, necrotizing soft tissue infections, nsti, necrotizing fasciitis, antibiotics, examination, physical, missed, physical examination

## Abstract

Necrotizing soft tissue infections (NSTIs) are deadly infections that can involve all tissue layers from the epidermis to the muscle. NSTIs can be difficult to diagnose, as skin manifestations are often absent early in the course of the disease, and they can be difficult to differentiate from simple cellulitis. Definitive diagnosis and treatment occur in the operating room. We present a case of a 65-year-old female who presented with lightheadedness and presyncope, with planned admission for an exacerbation of chronic obstructive pulmonary disease. Prior to admission, however, she was found to have an NSTI. The patient went to the operating room and had an extensive debridement followed by prolonged hospital stay. This case highlights the importance of a thorough physical examination in ill-appearing patients with non-specific symptoms, possible sepsis, and any alteration in mental status.

## Introduction

Necrotizing soft tissue infections (NSTIs) are uncommon and rapidly progressive skin and soft tissue infections that cause extensive tissue necrosis and systemic toxicity [1]. Common clinical findings include erythema, edema that extends beyond the visible erythema, severe pain out of proportion to examination findings, fever, crepitus, and skin bullae or necrosis [1-3]. The paucity of cutaneous findings early in the course of the disease, in addition to difficulty differentiating NSTIs from cellulitis and other superficial skin infections, makes NSTI an easily missed diagnosis [2,3]. Surgery is the definitive treatment for NSTI, and without it mortality approaches 100% [4]. We present a case of a 65-year-old female who presented with a chief complaint of lightheadedness and presyncope but no dermatologic complaints who was ultimately found to have an NSTI.

## Case presentation

A 65-year-old female with a past medical history of diabetes, hypertension, congestive heart failure (CHF), and chronic obstructive pulmonary disease (COPD) presented to the emergency department with a chief complaint of lightheadedness and near syncope over the last three days. Per the patient and her family, she had several falls over the last few days due to the lightheadedness. Associated symptoms included nausea, one episode of vomiting, mild shortness of breath, generalized weakness, and increasing somnolence, as well as burning on urination and mild lower pelvic pain over the last month. Review of systems was otherwise unremarkable.

Initial vital signs were notable for an oxygen saturation (SpO_2_) of 91%. Her SpO_2_ normalized on two liters per minute of oxygen via nasal cannula. On physical examination, the patient was slow to respond to questions, though she was alert and oriented to person, place, and time. Other pertinent findings included diffuse bilateral expiratory wheezes and mild suprapubic tenderness to palpation. On laboratory testing, the metabolic panel was notable for a glucose of 478 mg/dL, a sodium of 129 mmol/L, a chloride of 85.1 mmol/L, and an anion gap of 21.9 mmol/L. Complete blood count revealed a bandemia of 16% but was otherwise unremarkable. Urinalysis, troponin, coagulation studies, and thyroid-stimulating hormone were unremarkable. Venous blood gas showed a lactate of 1.85 but was otherwise normal and without acidemia. Chest x-ray showed pulmonary vascular congestion and stable cardiomegaly.

**Figure 1:**
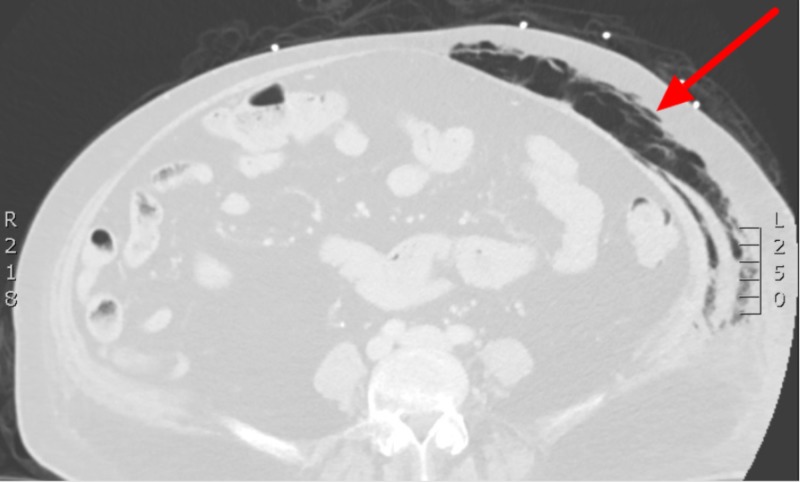
Transverse CT image showing soft tissue gas (red arrow) in the left anterior abdominal wall.

**Figure 2:**
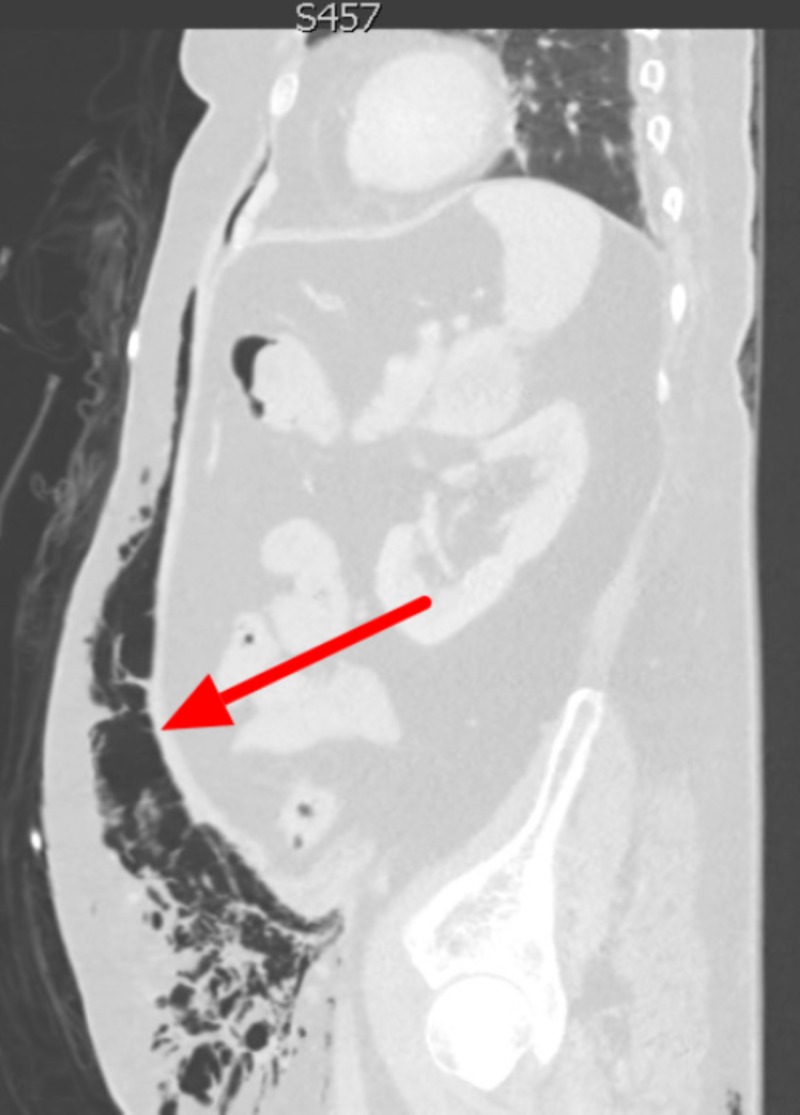
Sagittal CT image showing extensive soft tissue gas tracking along the patient’s entire left abdominal wall (red arrow) down to the thigh.

The patient was treated with nebulized albuterol-ipratropium two 0.3 mL doses, ceftriaxone 1 g intravenous (IV), azithromycin 500 mg IV, methylprednisolone 125 mg IV, and one liter of lactated ringers for suspected COPD exacerbation. The patient’s temperature later increased to 101.6°F, and the nurse notified the physician that while placing a Foley catheter, she noticed a red rash in the patient’s perineal region. On re-examination, the physician found crepitus in the lower abdomen/pelvis with associated tenderness, multiple fluctuant areas concerning for abscesses, and swelling/erythema/induration of the left labia extending to the proximal thigh. A computed tomography (CT) scan of the abdomen/pelvis with IV contrast revealed extensive subcutaneous and soft tissue gas tracking along the fascial planes of the left hemiabdomen, left greater than right labia, and left medial thigh concerning for NSTI (Figures 1, 2).

Based on examination and CT, clindamycin 600 mg, vancomycin 2 g, and acetaminophen 1,000 mg were administered intravenously in concern for NSTI, as well as a second liter of lactated ringers. General surgery was consulted and took the patient directly to the operating room. She underwent extensive debridement of all affected areas and had a complicated hospital course that lasted five months which consisted of multiple debridements and skin grafts.

## Discussion

NSTIs are uncommon diseases with a high mortality from rapid progression to septic shock and multi-organ failure [1,3]. The term NSTI is increasingly replacing the term “necrotizing fasciitis,” as the former refers not just to fascial involvement, but also muscle, skin, and surrounding tissues [1]. The incidence of NSTIs due to invasive group A streptococcal (GAS) infections in the United States is 0.4 per 100,000, and it is a disease seen predominantly in adults [2,5]. Events that predispose people to NSTIs include mild trauma, insect bites, drug reactions, illicit drug injections, perirectal abscesses, and surgical procedures. There is frequently an association between NSTIs and underlying chronic diseases such as diabetes, hypertension, CHF, obesity, renal insufficiency, cancer, malnutrition, arteriosclerosis, alcoholism, autoimmune disease, immunosuppression, and age over 60 years [6]. NSTIs most frequently involve the extremities, though the perineum is the most common spontaneous site of infection in which a direct cause is not found [3,6,7]. An NSTI that involves the perineum, genitals, or perianal region is specifically known as Fournier’s gangrene [1].

NSTIs are classified into types according to etiology. Type I is the most common form encountered. It is polymicrobial and involves a mixture of aerobic and anaerobic bacteria [2]. This type is usually seen in middle to older age individuals with comorbid conditions such as diabetes and other chronic diseases [2]. In one systematic review of nine studies involving 1,463 patients with necrotizing fasciitis, diabetes was a comorbidity in 44.5% of cases [8]. Our patient suffered from diabetes, and her NSTI fell into the type I category. Type II is the monomicrobial form, most commonly caused by GAS. It is often seen in young, immunocompetent patients with minor trauma, surgery, or intravenous drug abuse. Type III and IV NSTIs are extremely rare and refer to infections involving marine organisms and fungi, respectively [2].

NSTI is diagnosed based on history, physical examination, and operative therapy. In one study of 89 patients with necrotizing fasciitis, only 15% had an accurate diagnosis at the time of admission [3]. Therefore, a clinician must maintain a high clinical suspicion for NSTI to avoid misdiagnosis [6]. The most common findings in NSTIs include erythema, edema that extends beyond the visible erythema, severe pain out of proportion to exam findings, fever, crepitus, and skin bullae or necrosis [1-3]. In a recent meta-analysis including 23 studies and 5,982 patients, pooled sensitivity and specificity for fever were 46.0% (95% confidence interval [CI] 38.9%-53.2%) and 77% (95% CI 59.7%-88.1%), respectively, for hemorrhagic bullae 25.2% (95% CI 12.8%-43.7%) and 95.8% (95% CI 87.3%-98.7%), respectively, and for hypotension 21.0% (95% CI 9.4%-40.4%) and 97.7% (95% CI 91.4%-99.4%), respectively [9].

Laboratory findings in NSTIs are non-specific and only sometimes contribute to making the diagnosis. Leukocytosis and hyponatremia have been shown to improve sensitivity from clinical examination alone [1]. In addition, the Laboratory Risk Indicator for Necrotizing Fasciitis (LRINIC) score may aid in diagnosis. While it is poorly sensitive and should not be used to rule out NSTIs, a recent meta-analysis found a score greater than or equal to 8 to have a specificity of 94.9% [9]. With regard to imaging, plain radiographs may show increased soft-tissue thickness and opacity similar to cellulitis [2]. Subcutaneous air is a highly specific sign of NSTI. However, it is poorly sensitive and not seen on radiographs until the infection is advanced [2]. CT is considered the best initial imaging modality for NSTI, and findings include edema and asymmetrical thickening with or without enhancement of the deep fascial layers, as well as possible signs of cellulitis or myositis [2]. As with plain radiographs, the most specific finding for NSTI on CT is the presence of gas in the subcutaneous tissue [2]. There is emerging evidence to suggest the utility of bedside ultrasound in diagnosing NSTI. Ultrasound may show thickening and distortion of the deep fascia >4 mm, turbid fluid collections along the deep fascia and subcutaneous air [2,10]. Finally, magnetic resonance imaging provides excellent soft tissue images but is usually impractical in the emergent setting [1,2]. Despite the utility of CT in making a diagnosis, surgery should never be delayed while awaiting imaging results if clinical suspicion for NSTI is high [3,7].

Definitive findings of NSTI on surgical exploration include “dishwater” brown fluid, lack of tissue resistance to blunt finger dissection, lack of bleeding, and frank necrotic tissue. In equivocal cases where the diagnosis is uncertain, a surgeon may elect to perform a bedside “finger test” under local anesthesia. This is a bedside procedure in which a 2 cm incision is made down to the deep fascia. If any of the above findings are encountered, NSTI should be diagnosed and the patient taken to surgery [1,2].

Several studies have shown that early and aggressive debridement is the single most important treatment intervention for NSTIs. While awaiting surgical intervention, broad-spectrum antibiotics should be administered and supportive critical care provided [1,2,6]. Empiric antibiotic coverage should include a carbapenem or beta-lactam/beta-lactamase inhibitor to cover gram-negative pathogens and anaerobes, a methicillin-resistant Staphylococcus aureus active agent such as vancomycin or daptomycin, and clindamycin due to its inhibition of protein synthesis and potential antitoxin properties against GAS [1,11].

The case of our 65-year-old female patient is unique in that her overall presentation was non-specific, with a chief complaint of lightheadedness and presyncope. Her presentation was initially attributed to hyperglycemia, dehydration, and a COPD exacerbation. It was not until the patient spiked a fever and the nurse noticed an abnormal skin finding while placing a Foley catheter that the diagnosis was re-evaluated. A visual examination of the abdomen performed on initial physical examination would have revealed erythema along the patient’s lower abdomen that would have then prompted further visualization of her pelvis and groin. In addition, the patient’s family said that the patient was increasingly somnolent, and despite being completely alert and oriented on examination, she was slow to respond to questions. In the context of her dysuria and mild suprapubic tenderness to palpation, this was initially attributed to possible urinary tract infection. However, the patient should have been re-examined for another infectious source after the urinalysis came back unremarkable.

This case emphasizes the critical role of physical examination in evaluating a patient. In an era where technology and special testing are more frequently relied upon for accurate diagnosis, the clinician must remember that such tests should serve as a supplement rather than a replacement for basic medical skills [12,13]. While a focused physical examination is appropriate for many patient complaints, a more comprehensive exam is warranted in any elderly patient with multiple non-specific complaints or even mild alteration in mental status. A clinician should maintain a high clinical suspicion for NSTI in such patients, as they may present with systemic illness and encephalopathy alone [1,2]. These patients should have a thorough skin examination to look for Fournier’s gangrene, decubitus ulcers, or any other hidden source of infection [3]. In one cross-sectional study in which physicians were surveyed on physical examination oversights that may have led to a missed or delayed diagnosis, 63% said the oversight was caused by a failure to perform the physical examination. Had more attention been given to examining the skin, as well as the abdomen, groin, and genitourinary area, the reported oversights could have been reduced by half [14].

## Conclusions

NSTIs are aggressive diseases that can quickly lead to death if missed. A thorough skin examination and a high index of suspicion are important in any elderly patient with multiple non-specific complaints or any alteration in mental status. In an era where clinical skills are increasingly being replaced by technology and special tests, one must remember that the history and physical examination serve as the basis for guiding further testing and management.
